# Arytenectomy for Roaring in the Horse1Read before the Thirty-second Annual Convention of the U. S. Veterinary Medical Association, Des Moines, Iowa, Sept. 11, 1895.

**Published:** 1895-12

**Authors:** S. J. J. Harger

**Affiliations:** Professor of Veterinary Anatomy and Zootechnics, University of Pennsylvania


### ARYTENECTOMY FOR ROARING IN THE HORSE.[1]

By DR. S. J. J. HARGER,
PROFESSOR OF VETERINARY ANATOMY AND ZOOTECHNICS, UNIVERSITY OF PENNSYLVANIA.

SINCE writing on this subject of arytenectomy for roaring, as well as reporting some cases on previous occasions, I have been still more anxious to determine the value of this operation. Hoping to have a much more valuable and exhaustive paper than the one I now present to you, I sent to the veterinarians of Philadelphia and vicinity a number of circulars explaining my motives and soliciting cases of roaring which I agreed to operate on for a nominal fee, simply covering expenses. If the operation should prove to become successful it will be remunerative both to the veterinarian and to the horse-owner; if it be not practical it is necessary that the profession should know. Unfortunately, the number of cases which I was in this manner able to secure fell short of my anticipations. In some cases I have been, as yet, unable to secure any reports; in others the time that has elapsed since the operation is too short to allow

[1] Read before the Thirty-second Annual Convention of the U. S. Veterinary Medical Association, Des Moines, Iowa, Sept. 11, 1895.

me to draw any conclusions, or even to ascertain the actual condition of the horse. However, I will give a synoptical *résumé* of some cases with the termination ascertained.

CASE I. English thoroughbred stallion, seven years of age, sired by Joe Hooker. Roaring decided. The horse could not run a half-mile in 1.05, nor even a fair quarter without falling down from suffocation. Assisted by Dr. Charles Lintz, of Chester, Pa., I operated in April, 1895. Did well during June and July. August 1st was ready for a race, when he had an attack of colic and for a few days had a discharge from the nostrils, from a part of the colic-drench entering the trachea. Since the operation the horse ran a half-mile in 51 seconds "and did not take a long breath." He also ran three-fourths of a mile—first quarter in 37 seconds, second quarter in 27½, and third quarter in 25 seconds, and did not roar. Owner says that it is an entire cure.

CASE II. Black mare, twelve years of age. Heredity and cause unknown. Symptoms first recognized in 1888 and gradually increased, and in the fall of 1894 the mare was unable to work. Used for general purposes. Operated December, 1894. No complications followed. Since the operation, according to the information given me by the owner and veterinarian, Dr. Hogg, who assisted me, the mare has performed almost all kinds of work without making any noise, and is pronounced sound. At present she is working on the tread-power of a threshing-machine.

CASES III., IV., and V. Two of these three cases were common horses, while the third was fairly well-bred and possessed some speed. Chronic roarers; heredity unknown, as nearly as I can now recollect. Operated in the early part of November, 1894, at the infirmary of Dr. H. H. Choate, Lewiston, Maine. I have no detailed history of these horses subsequent to the operation, but Dr. Choate, who assisted me and treated the patients afterward, said, in a communication to me, that the three "had made a *complete recovery*."

CASE VI. Bay mare, standard bred. Suffered from "distemper" in December, 1894, and has roared ever since. She could not be driven a block on a slow jog without roaring badly, and was useless for any kind of work. Operated April, 1895. She still has, though seldom, a slight cough when drinking water, which sometimes runs through the nostrils, though this does not inconvenience the animal. She can now go at the rate of ten miles an hour without making any noise. When driven to her speed, a 2.30 gait, she makes some noise, though not very much. She is still improving, and the owner, a physician, feels confident that she will ultimately recover. To use his words, "anyone having a horse similarly affected should apply . . . . for the operation of arytenectómy."

CASES VII., VIII., IX., X., XI., XII. These cases, which I assume the liberty of reporting, were reported to me by Dr. H. H. Choate. They were operated on by him since November 1st. Out of the six, he informs me, four made a complete recovery and two make a little noise when severely exercised. I have no other details of these cases at present.

CASE XIII. Bay mare, aged. Common bred. History indefinite. Symptoms marked; roared severely after a gallop of two hundred yards or when worked to a cart. Operation, March, 1895. At present she makes a slight noise when driven at full speed, especially uphill, and the noise ceases as soon as she is stopped. She can be driven as fast as her speed will permit and is now *serviceable*.

CASE XIV. Operation at the same time as cases three, four, and five. This animal is *somewhat improved*.

CASE XV. Black gelding, fairly well-bred. Was affected with strangles complicated with pneumonia(?) during the previous June. The symptoms were not very severe. Operation, February, 1895, assisted by Dr. M. E. Conard. He makes no noise when "getting his breath," but when the lungs are filled with air he seems have trouble in expelling it, and at times coughs. Owner says he is *very much improved*, but not cured.

CASE XVI. Gray mare, well bred; six years of age; disease existing for three years and resulting from distemper. Symptoms irregular: at times, any amount of exercise would not make him roar, while at other times exercise on the halter was accompanied with considerable noise. Operation, November, 1895, assisted by Dr. Bland, of Waterbury, Conn. After opening the larynx, I found that the movements of the vocal cords were normal and equal on both sides, a condition which was not the most favorable one for the success of the operation. At present there is not much change in the animal. She is serviceable and is driven every day. When limited as to the bulk of her food and water she does not make any noise.

CASE XVII. Gray gelding, coach-horse. Operation, May, 1894, assisted by Dr. G. H. Berns, of Brooklyn, N. Y. Patient did exceedingly well for three or four weeks after the operation, and the wound healed kindly. Between the third and fourth weeks, when the wound was almost closed, he was exercised to the halter, and trotted twice up and down Adams-Street hill. His respirations were normal, and no noise could be detected. Some three or four days later he began to cough; would cough eight or ten times in succession, and after each paroxysm the respirations would be quite raspy for some fifteen or twenty minutes; no loss of appetite, no fever, and none of the usual symptoms of laryngitis or bronchitis were observed. The coughing-spells became more frequent and severe; the respirations gradually became more noisy. His condition grew gradually worse, until one morning, some six weeks after the operation, when his breathing became so distressed that it was necessary to perform tracheotomy. The *tube* was left in for a week or ten days. The breathing was normal, but the moment the tube was taken out and the opening closed he would roar loud enough to be heard at some considerable distance. Two months after the operation the animal was destroyed.

*Autopsy.* Original incision through the cricoid cartilage completely healed and divided portions in direct apposition; interior of larynx free from all granulations or foreign substances; mucous membrane in a perfectly normal condition, and the cicatrix could only be detected on careful examination by a slightly puckered condition of the membranes at the point where the left arytenoid cartilage had been.

CASE XVIII. Bay gelding. History indefinite. Operation, June, 1895. The animal has not been sufficiently exercised to determine how much he roars. He has some difficulty in swallowing, with a disagreeable cough when exercised. I have reopened the larynx by cutting through the crico trachealis ligament (the horse standing with a twitch) and found the seat of operation entirely healed. Termination unsatisfactory.

CASE XIX. Gray mare, standard bred. Had pneumonia five years ago, and roared ever since. When I saw her she was in poor condition, the respirations were very loud both on inspirations and expirations, even threatening suffocation, and the subject was not such as to allow one to draw an accurate conclusion as to the result of an operation. I did not see the mare afterward, but she died about five days after the operation.

In some instances I have met with complications; slight pneumonia in two cases, from which the animals recovered. In others there is a slight cough with regurgitation of water through the nose in drinking. The cause of both in Case XIX. I do not know. There was no autopsy, but the larynx was in good condition.

I will not here offer any new suggestions in the *modus operandi*, except in suturing the mucous membrane, which is difficult and tedious. In one case I used perforated shot and silver wire. I obtained a closer apposition of the edges of the incised mucous membrane, and the cicatrix was smaller Silk saturated with blood is slippery, and the knot will loosen before completed. The shot may drop out through the incision in the larynx or may pass down into the bronchi. Even in the latter case I would not anticipate any serious trouble.

To recapitulate: From these 19 cases the following results may be deduced: 10 cases are admitted to be cured; 1 is very much improved; 2 roar only when severely exercised; 2 improved and serviceable; 1 no difference and serviceable; paralysis of the laryngeal muscles doubtful; 2 died; 1 termination unsatisfactory.

